# Lunacy and Pauperism

**Published:** 1896-03-21

**Authors:** 


### Modern Sociology.

### LUNACY AND PAUPERISM.

Of all the changes and chances of human life, the saddest, perhaps, are those which operate to deprive a man of the means or capacity to earn his living, and which naturally tend towards pauperism, that last, and, to the self-respecting man, most unwelcome stage of mortal existence. Even in these utilitarian days, when hard matter-of-fact ideas have largely superseded sentiment, the honest old-world feelings of revulsion against the degradation is hard to die, and only those among us who are utterly callous, inured by hardship to deadly indifference, can endure the stigma without a pang of shameful sorrow, and an aching heart. The feeling is altogether commendable, and is deserving of every kind of encouragement, apart from the more sordid and very obvious reasons which exist as barriers to the too free dispensation of parochial relief. Upon the broadest and most comprehensive ground, anything which tends to break down the spirit of personal self-respect which is so marked a feature of our social characteristics, or which adds to the heavy burden of our social obligations, is to be deplored and avoided. In view of these considerations, it seems strange that the calamity of lunacy should be allowed to be the direct means of forcing into pauperism thousands of people who are not in need of pecuniary aid from the relieving officer, and who are driven to resort to him only because he is the official to whom the administration of the lunacy law in its initiatory stages is entrusted. It would be difficult to suggest a better medium for the purpose. The officers have the command of all necessary appliances and means of transport; they are accustomed to the control of refractory and unmanageable subjects, and their services are easily available at all hours; whilst the care and charge of the many lunatics who are known paupers is an integral part of their ordinary duties. In practice, the discrimination between lunatics who really are paupers and those who are not has been found a matter of some difficulty, owing to the necessity for prompt action in the preliminary proceedings, and, therefore, it has been enacted that all persons sent to asylums under summary detention orders should be classified as paupers until the fact was established that they were entitled to be dealt with as private patients. The cases open to investigation on this point are those of (1) lunatics who are not, or who have not been previously known as paupers, and who are not under proper care and control; and (2) lunatics who are not paupers wandering at large. The detention order should in all cases specify the circumstances under which the lunatics are found—whether belonging to either of the two classes mentioned, or whether (3) they are "paupers in receipt of relief." Now, inasmuch as it is always necessary to call in medical assistance at the cost of the parish—a step which in itself technically involves the granting of parochial relief—it is clear that all cases may be, and, indeed, often are, included in the third category, without reference to other circumstances, with the result that no subsequent inquiry as to classification takes place, and the patient remains in the asylum as a pauper lunatic. Even, however, if due inquiry is made, the result is the same unless the patient or his friends are able to pay the cost of his maintenance in a private institution, the amount of which, in the large majority of cases, is simply prohibitive. It is no matter that all the charges incurred by the parish are refunded to the uttermost farthing. The patient is pauperised simply by the advance of temporary relief, and in this respect the repayment counts for nothing. Nor is this all. A man, whose wife is an inmate of a public asylum under these circumstances, is himself pauperised, and incurs all the attendant disabilities, including disfranchisement. He is to all intents and purposes as much a pauper as any inmate of the parish workhouse, although he may be in the actual receipt of a competent income, paying his way in the world, maintaining his family in comfort, and actually defraying the expenses of his wife's detention and treatment in the asylum. These are circumstances which appear to us to call for comment and inquiry. Section 18 of the Lunacy Act, 1890, defines a pauper to be one "in receipt of relief or in such circumstances as to require relief for his proper care." Surely the inmate of an asylum, in respect of whom all charges are paid from his own resources, or by relatives or friends, does not come within this limitation, and ought not to be classified as a pauper merely because the poor-law authorities provide the channel through which the payments to the asylum are made. And, surely, no man should be disfranchised simply because of the terrible affliction which has fallen upon him through the insanity of his wife, and so be subjected to a very real grievance, and a needless aggravation of his trouble. A complete remedy for this great wrong can probably only be effected by legislation; but we venture to assert that statutory powers already exist which would go far to ameliorate it if put in force. The Lunacy Act renders it expressly compulsory upon the local authorities therein defined to provide asylum accommodation for the pauper lunatics within their jurisdiction; and it also confers upon such local authorities power to make similar provision for private patients upon such terms as to payment and accommodation as they may think fit. Apparently this last-mentioned power is permissive only, but in view of the fact that the 13th, 15th, and 16th sections of the Act distinctly authorise any justice to order the detention of insane persons who are not paupers in any institution for lunatics, it would appear that there is an implied obligation on the local authority to make provision for their reception. Apart from this, however, the question is one of public right. Let it be remembered that the local authorities are not arbitrary bodies, but are the elected representatives of the whole community. They have, as we have seen, the power to provide accommodation in their asylums upon terms and conditions which, without any public loss, would at the same time entail no needless expense upon the individual. A charge very slightly in excess of that now made in respect of pauper patients would be sufficient for all purposes, and would be within the means of many to whom the cost of detention in private asylums is altogether prohibitive. The exercise of these powers by the local authorities would go far to remove the public grievance we have indicated; the failure to do so deprives many individual members of the community of a statutory privilege to which they are entitled, and operates as a direct incentive to the increase of pauperism.

